# Machine Learning Analysis of Gaze Data for Enhanced Precision in Diagnosing Oral Mucosal Diseases

**DOI:** 10.3390/jcm13010136

**Published:** 2023-12-26

**Authors:** Shuji Uchida, Shin-ichiro Hiraoka, Kohei Kawamura, Katsuya Sakamoto, Ryo Akiyama, Susumu Tanaka

**Affiliations:** Department of Oral and Maxillofacial Surgery, Graduate School of Dentistry, Osaka University, 1-8 Yamada-Oka, Suita 565-0871, Osaka, Japan

**Keywords:** oral mucosal diseases, visual examination, gaze data, diagnostic algorithm, support vector machines, heatmap analysis, intraoral photographs

## Abstract

The diagnosis of oral mucosal diseases is a significant challenge due to their diverse differential characteristics. Risk assessment of lesions by visual examination is a complex process due to the lack of definitive guidelines. This study aimed to improve this process by creating a diagnostic algorithm using gaze data acquired during oral mucosal disease examinations. A total of 78 dentists were included in this study. Tobii Pro Nano^®^ (Tobii Technology) was used to acquire gaze data during clinical photographic visual examinations. Advanced analysis tools such as support vector machines and heatmaps were used to visualize the gazing tendencies of a group of skilled oral surgeons, focusing on the number of gazes per region and the gazing time ratios. The preliminary findings showed the possibility of visualizing gazing tendencies and identifying areas of importance for diagnosis. The classification of intraoral photographs based on gross features revealed the existence of an optimal examination method for each category and diagnostically significant areas. This novel approach to analyzing gaze data has the potential to refine diagnostic techniques and increase both accuracy and efficiency.

## 1. Introduction

Oral cancer is relatively rare, accounting for less than 5% of all carcinomas. More than 300,000 new cases of oral cancer are reported globally each year [1,2,3]. Unlike gastrointestinal cancers, oral mucosal diseases, including malignant tumors and precancerous lesions, can be visually detected without complex devices such as endoscopes. Regular oral health checkups facilitate the early detection of such lesions [3], making timely therapeutic intervention possible and improving survival rates [4,5]. However, many oral mucosal diseases are diagnosed at an advanced stage because of inadequate risk assessment and delayed referrals to higher-level medical facilities [6]. Thus, effective screening methods for oral mucosal diseases are needed. Such methods enable appropriate risk assessment before the disease advances. Objective assessment of the oral cavity through visual examination and palpation is indispensable. Besides palpation, early tongue cancer hardness assessments have been conducted using ultrasonography [7]. However, accurate assessments remain challenging for clinicians due to the limited number of cases and evaluation criteria. For visual examination, numerous atlases visually present features associated with oral mucosal diseases. However, the vast diversity of diseases listed for each differential diagnosis results in scattered cases where the diagnosis is complicated [8].

Therefore, developing educational tools for visual examination is essential for improving diagnostic skills for oral mucosal diseases. However, no studies have evaluated the suitability of examination methods based on visual examination for pathological pictures [9,10,11]. The COVID-19 pandemic has increased the urgency for online medical consultations [12,13]. Consequently, there is a need to determine the most effective examination methods for accurately diagnosing oral mucosal diseases via monitors. Thus, this study aimed to create a novel diagnostic algorithm for oral mucosal diseases using gaze data captured during monitor-based examinations and analyzing the diagnostic techniques and tendencies of experienced clinicians.

## 2. Materials and Methods

### 2.1. Study Design and Target Selection

A total of 78 dentists were included in this study. The participants were divided into two groups: skilled oral surgeons, who are dentists with more than eight years of clinical experience, and general dentists, who primarily practice outside of oral surgery (Table 1) [1]. Eye gaze data were obtained from the participants. Skilled oral surgeons demonstrated a higher overall correct response rate (85.8%) than general dentists (49.70%). The reason for including these two groups was to examine potential disparities in the diagnostic gaze patterns of dentists with different specialties and levels of experience since this study focused on determining differences in diagnostic strategies between experienced oral surgeons and general dentists using gaze data analysis.

### 2.2. Apparatus Selection

**Figure 1:**
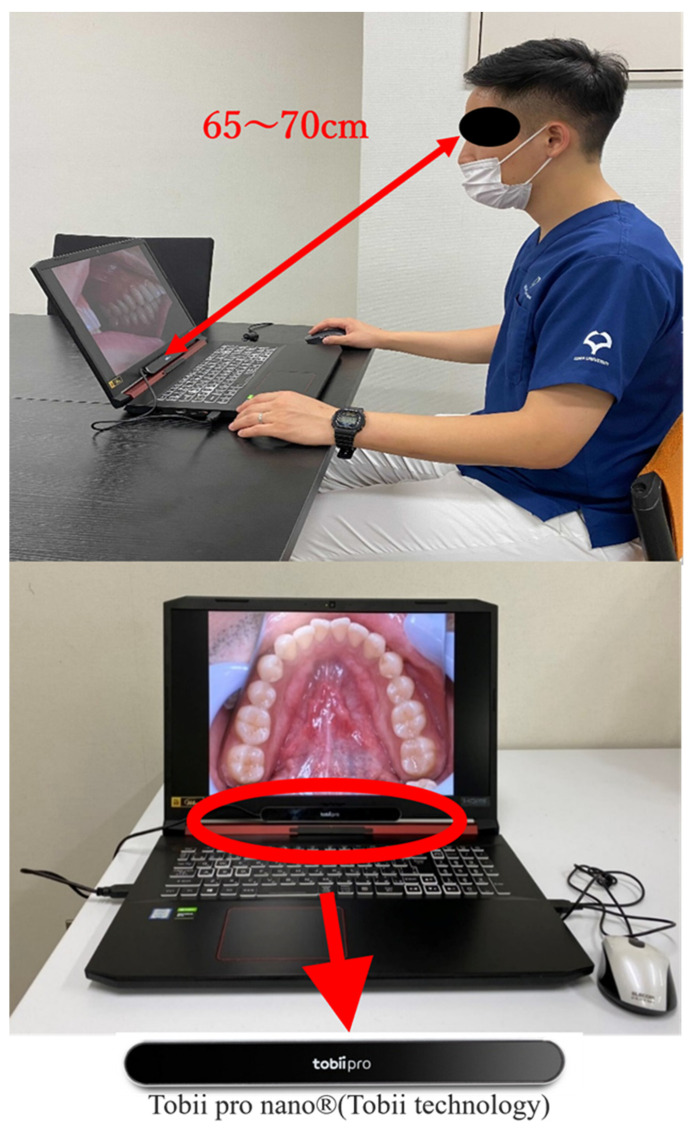
Tobii Pro Nano^®^ (Tobii Technology) eye tracker setup for gaze data acquisition.

Eye gaze data were recorded using a Tobii Pro Nano^®^ (Tobii Technology, Inc., Tokyo, Japan) (Figure 1). This device was chosen because of its precision in capturing gaze data, enabling us to pinpoint focus areas during visual examinations with high resolution [2]. Before the recording of an individual’s eye movement data acquisition begins, there is a process called calibration, which is used to obtain the geometric features of the subject’s eyes and accurately calculate the viewpoint. As the subject gazes at a specific point displayed on the monitor (calibration point), a light reflection pattern is generated on the subject’s cornea using a built-in near-infrared LED and acquired by the eye tracking camera along with geometric features (location and shape of the central fossa, information about light refraction and reflection characteristics) The results are then used to create an anatomical 3D eye model. It is possible to create an anatomical 3D eye model and calculate individual viewpoints.

### 2.3. Methods of Acquiring and Analyzing Gaze Data

Upon calibration of the Tobii Pro Nano^®^, at eight points on the display, various intraoral photographs were randomly exhibited on a 17.3” full HD display (1920 × 1080) to simulate a real-world diagnostic environment. The participants were positioned at a distance of 65–70 cm from the screen to mimic a typical viewing distance in clinical practice. The participants were asked to examine each image freely, provide a clinical diagnosis, and name the potential disease. All procedures were approved by the Ethics Review Committee of the Osaka University Graduate School of Dentistry and Faculty of Dentistry Hospital (approval number: H29-E35-1). The gaze coordinate data for each photograph and the accuracy of the diagnostic results were then analyzed using Tobii Pro Lab^®^ gaze analysis software version 1.217 (Tobii Technology Corporation, Tokyo, Japan). Any gaze points with a duration of less than 250 ms were excluded to ensure a reliable analysis of the gaze data, following established guidelines that an average gaze time of 250–300 ms is necessary for visual information to be processed by the brain [14,15].

### 2.4. Selection and Classification of Intraoral Photographs

**Figure 2:**
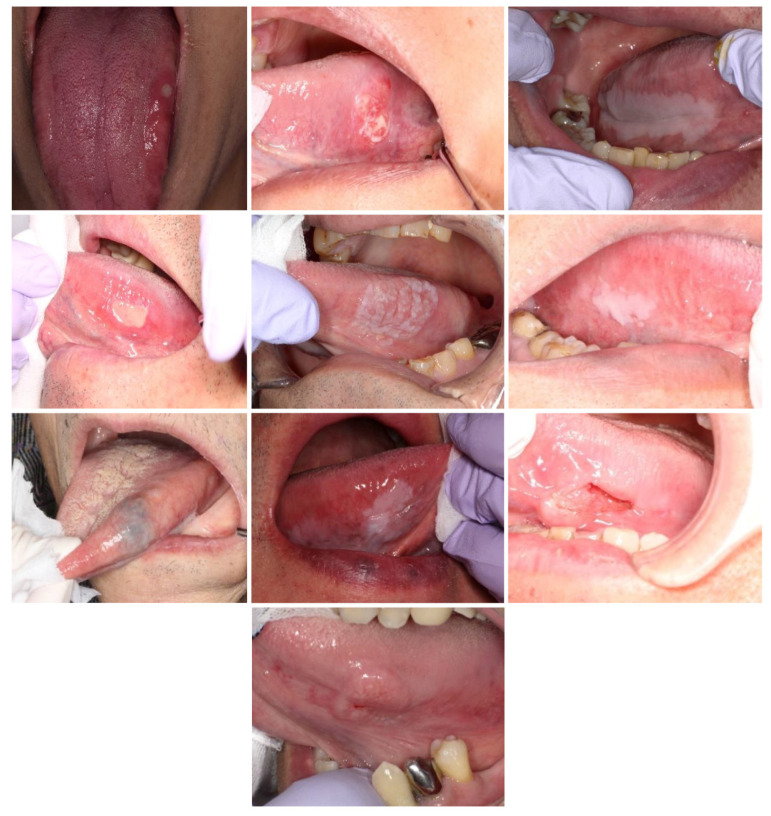
The intraoral photographs selected for gaze data acquisition.

A total of 30 intraoral photographs were employed, each depicting one of the following five categories: malignancy, stomatitis, leukoplakia, benign tumor, and normal conditions. The photograph selection was subject to three conditions: the lesion had to be present on the tongue, it had to be large enough to be statistically processable (occupying more than 2% of the display area), and it had to demonstrate an unbiased correct response rate. After applying these criteria, ten photographs were selected for this study (Figure 2).

### 2.5. Visualization of Clinicians’ Diagnostic Evidence Using Gaze Data

The distribution of gaze points and areas using heatmaps and support vector machines (SVMs) was visualized to understand the visual strategies employed by clinicians during diagnosis [16].

### 2.6. Delineation of the Internal and External Areas of the Lesion

**Figure 3:**
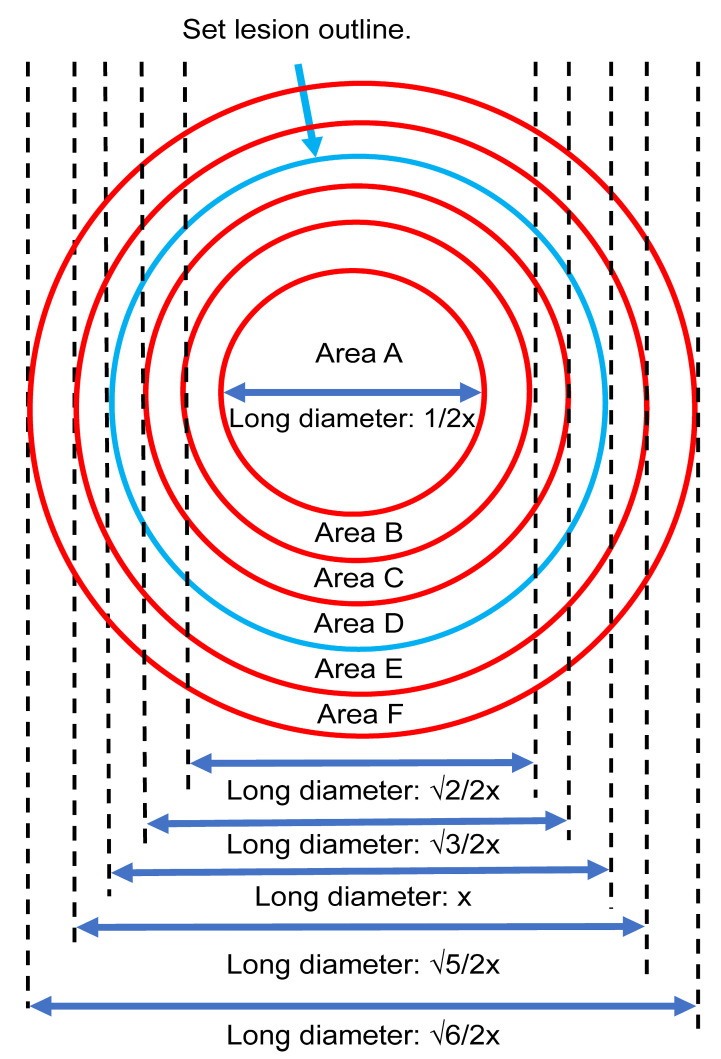
The internal (Areas A–D) and external (Areas E and F) regions of the lesion based on multiple clinicians’ perspectives.

The lesion area and the surrounding region were divided into six equal areas (A–F) based on multiple clinicians’ perspectives to facilitate further analysis. The interior of the lesion was labeled as areas A–D, while the periphery was labeled as area E and F (Figure 3).

### 2.7. Statistical Studies on Optimal Screening Methods and Areas of Diagnostic Evidence

In our analysis, we focused on quantitatively evaluating the gaze behaviors of participants during the examination of oral mucosal diseases. Specifically, we calculated the number of gazes and the gaze time ratios for each region of interest within this study. This allowed us to comprehensively assess how participants’ attention was distributed across different areas during their diagnostic process. To compare these gaze metrics, we employed an Analysis of Variance (ANOVA) approach. This statistical method is particularly effective in assessing the differences across multiple groups, enabling us to discern any significant variations in gaze patterns among the different clinician groups involved in our study. In addition to the ANOVA, we also implemented post hoc tests with Bonferroni correction. The Bonferroni correction is a stringent method of adjusting for multiple comparisons, thereby reducing the likelihood of false-positive results. This approach ensured that our findings remained robust even when comparing multiple regions and groups. For comparisons within the same group, we used the paired *t*-test. For comparisons between different groups, we utilized Student’s *t*-test. All statistical analyses were performed using EZR version 1.54 (Saitama Medical Center, Jichi. Medical University, Saitama, Japan) [17]. The threshold for statistical significance is set at *p* < 0.05.

## 3. Results

### 3.1. Visualization of Diagnostic Evidence Using Gaze Data

**Figure 4:**
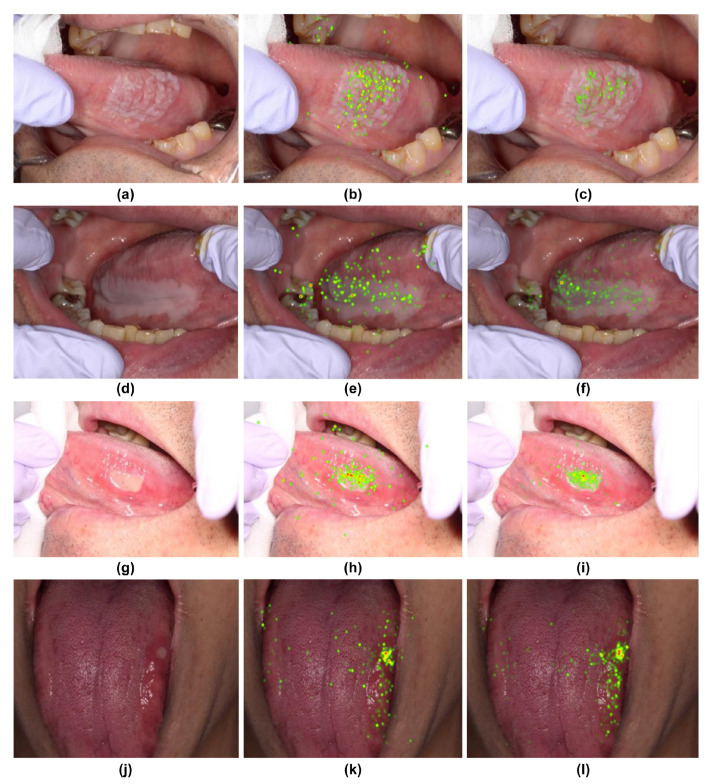
Heatmap comparison of gaze patterns during medical examinations. (**a**,**d**,**g**,**j**) Original images. (**b**,**e**,**h**,**k**) Heatmap based on gaze data from skilled oral surgeons. (**c**,**f**,**i**,**l**) Heatmap based on gaze data from general dentists.

Gaze data from the two groups revealed distinct differences when visualized through heatmaps and SVM. Among the ten photographs used, four showed a common feature: the skilled oral surgeons had a wider distribution of gaze points, including the margins of the lesion, whereas the general dentists focused more on the center of the lesion (Figure 4). This was consistent across four different types of lesions depicted in the photographs (Figure 4).

**Figure 5:**
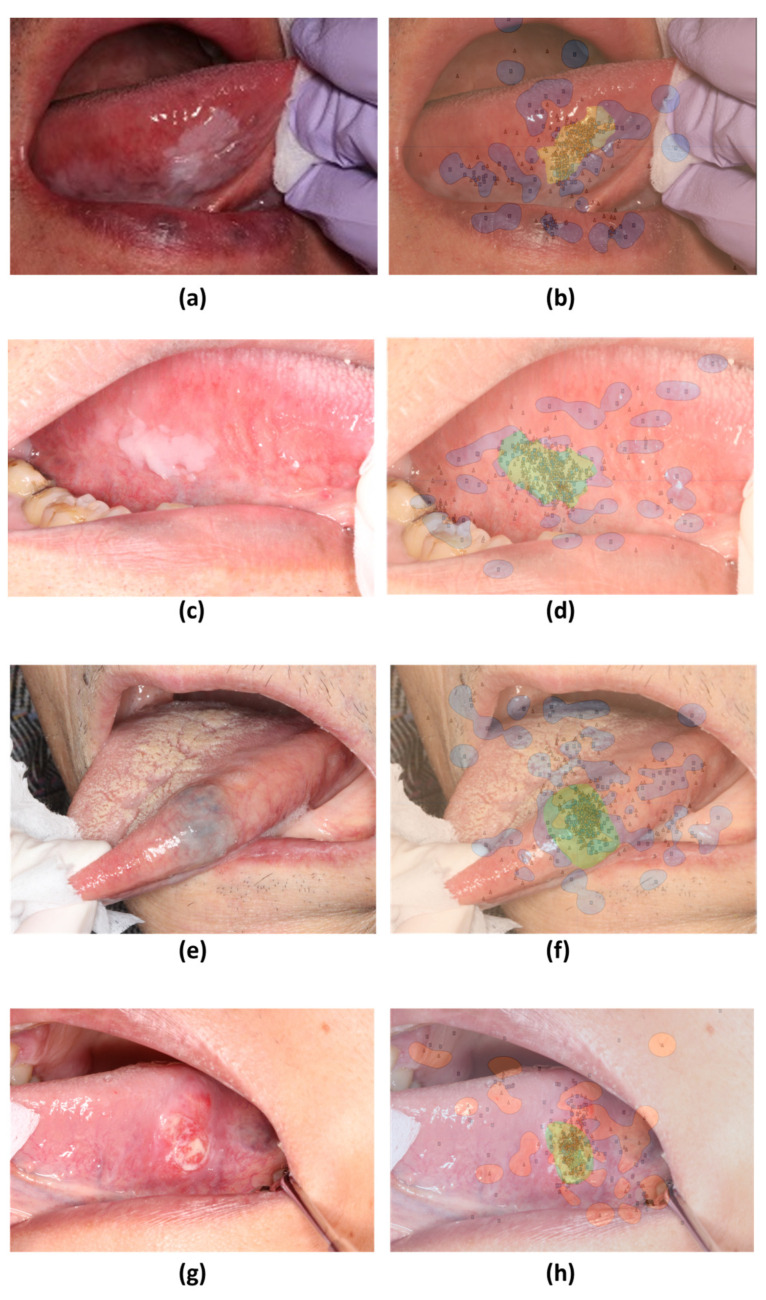
Decision boundaries created using support vector machines (SVM) based on gaze patterns during medical examinations. (**a**,**c**,**e**,**g**) Original images. (**b**,**d**,**f**,**h**) SVM decision boundaries based on gaze data from skilled oral surgeons and general dentists.

**Figure 6:**
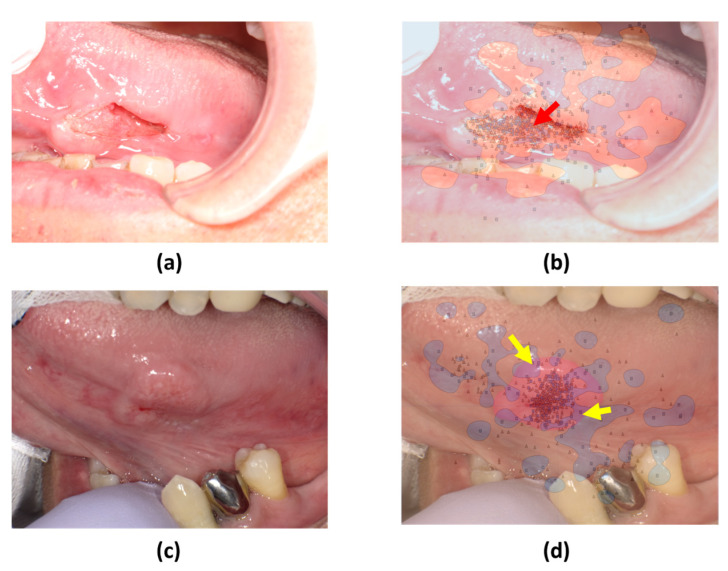
SVM comparison of gaze patterns. (**a**,**c**) Original images of tumors with distinct surface characteristics. (**b**,**d**) SVM decision boundaries based on gaze data from skilled oral surgeons and general dentists.

The SVM decision boundaries for the remaining six photographs split them into two groups (Figure 5 and Figure 6). The first group (Figure 5) demonstrated a tendency for skilled oral surgeons to focus on the lesion margins and outer areas. The second group (Figure 6) showed an exception to this trend. Oral surgeons’ gaze points tended to be concentrated on the lesion’s inner side.

### 3.2. Statistical Analysis of Optimal Screening Methods and Areas of Diagnostic Evidence

**Figure 7:**
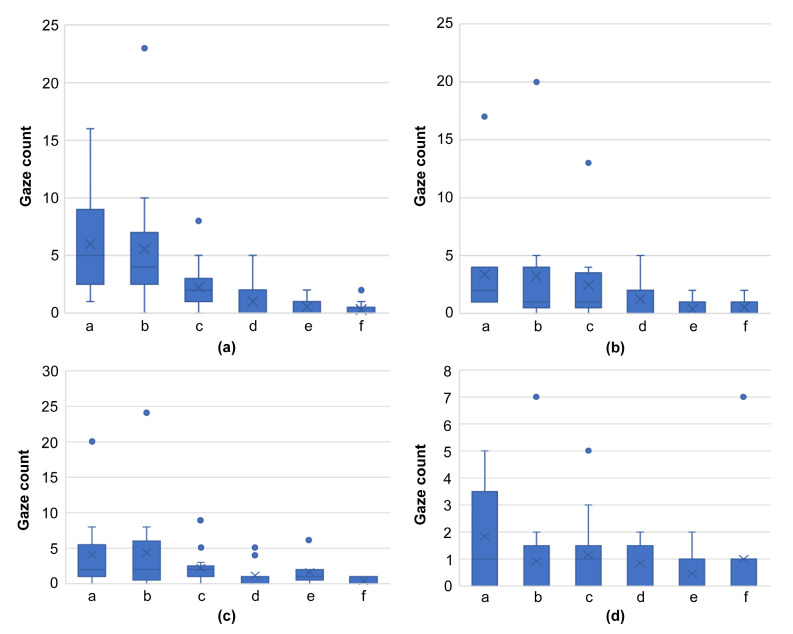
Graph comparing the number of gazes per domain for skilled oral surgeons, using images from Figure 4 ((**a**): Figure 4a, (**b**): Figure 4d, (**c**): Figure 4g, (**d**): Figure 4j). This graph reveals the absence of a significant difference in gaze frequency across different regions, indicating the broadened focus of skilled oral surgeons on all areas of the lesion.

**Figure 8:**
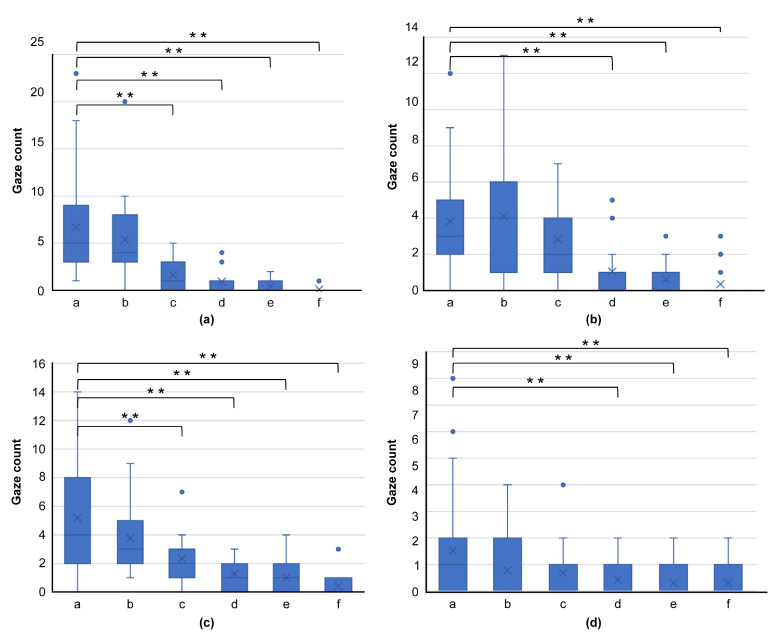
Graph comparing gaze frequency per domain for general dentists, using images from Figure 4 ((**a**): Figure 4a, (**b**): Figure 4d, (**c**): Figure 4g, (**d**): Figure 4j). This graph shows clear differentiation, with general dentists primarily focusing on region “a,” highlighting their tendency to concentrate on the center of the lesion. (** *p* < 0.01).

In terms of gaze frequency per region (a–f) for skilled oral surgeons (Figure 7) and general dentists (Figure 8), no significant difference in gaze frequency was observed among the oral surgeons across different regions. However, general dentists showed clear differentiation, focusing more on region “a.”

**Figure 9:**
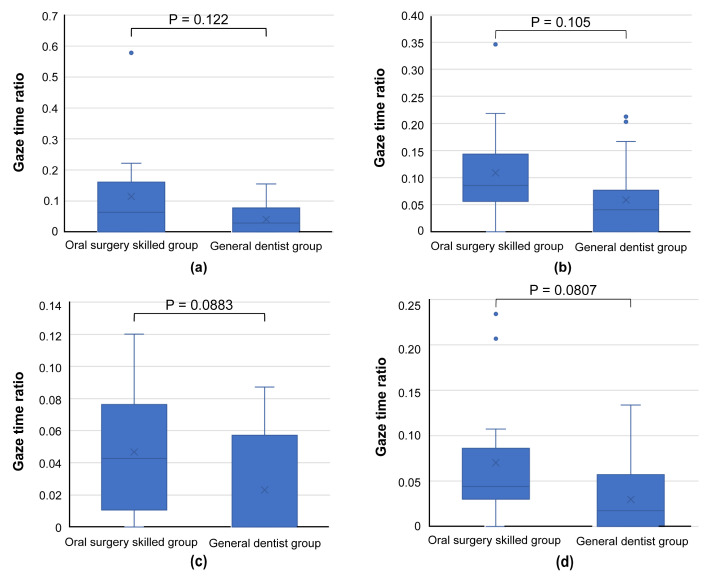
Graph comparing gaze time ratios for areas bd, e, and f, as presented in the images of Figure 5 ((**a**): Figure 5a, (**b**): Figure 5c, (**c**): Figure 5e, (**d**): Figure 5g). Skilled oral surgeons generally spend more time than general dentists examining these regions, further supporting the broader focus strategy of experienced oral surgeons.

A comparison of the gaze time ratios for photographs showing a common trend in SVM revealed a tendency for skilled oral surgeons to spend more time examining the same region than general dentists (Figure 9).

**Figure 10:**
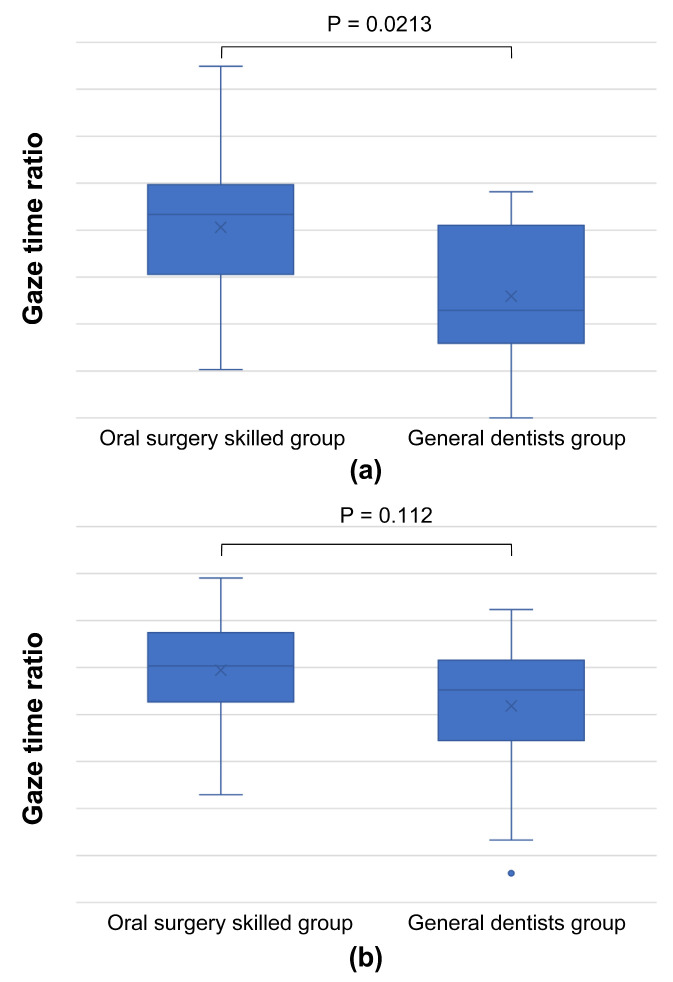
Graph comparing gaze time ratios in the inner lesion area, using images from Figure 6 ((**a**): Figure 6a, (**b**): Figure 6c). This graph indicates that skilled oral surgeons have a higher gaze time ratio in the inner region of the lesion, suggesting that, under certain conditions, their focus can be more centrally concentrated, contrary to the general trend.

Lastly, two images that showed a common trend in SVM (Figure 6a,c) demonstrated a higher gaze time ratio in the inner lesion region for skilled oral surgeons, consistent with the results visualized by SVM (Figure 10).

**Figure 11:**
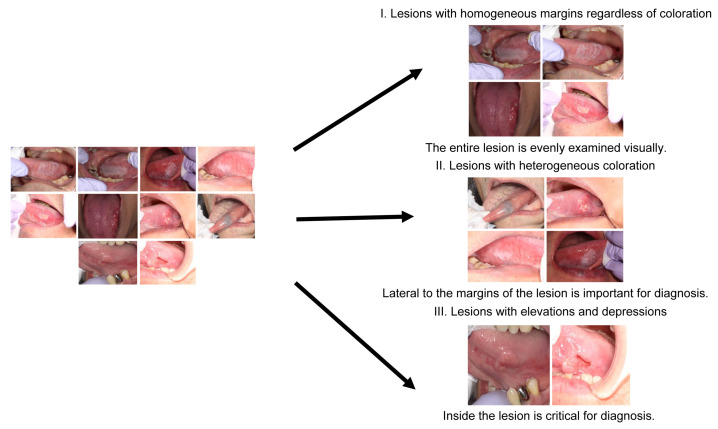
Graph depicting the relationship between gross features, screening methods, and diagnostic trigger areas.

Our results showed that skilled oral surgeons and general dentists exhibit different gaze patterns when diagnosing oral conditions. Skilled oral surgeons’ gaze patterns tended to cover a wider area, including the lesion margins, whereas general dentists primarily focused on the center of the lesion. This observation held true for different types of lesions and was supported by heatmap and SVM visualizations. As depicted in Figure 11, a discernible relationship exists between gross features, screening methods, and diagnostic trigger areas. Figure 11 is a comprehensive graph that demonstrates this relationship. It suggests that the diagnostic strategy, informed by the gross features of the lesion, influences the regions of focus or “diagnostic trigger areas” for both skilled oral surgeons and general dentists. This representation highlights the importance of training in broadening the areas of attention during the diagnostic process.

## 4. Discussion

This study explores the utility of gaze data analysis in improving diagnostic capabilities, with particular emphasis on oral mucosal diseases. The novelty of this study is that it targets intraoral photographs that lack standardization in their imaging methods, unlike conventional studies focusing on uniform imaging methods such as CT, MRI, mammography, and panoramic radiographs [18,19,20]. Our approach could be useful in primary dental institutions and for the future implementation of artificial intelligence medical equipment and online medical care.

The study design considered varying levels of clinical experience, dividing the subjects into initial trainees, oral surgeons, and general dentists. Skilled oral surgeons with more than eight years of clinical experience demonstrated superior diagnostic accuracy for all six diseases studied.

Furthermore, an innovative approach of classifying intraoral photographs based on gross features rather than the confirmed disease was introduced, which provided insights into the optimal examination methods and identification of key areas for diagnosis [21,22,23,24,25]. These gross features were categorized as uniform margins regardless of color tone (α), uneven color tone at the margins (β), and the presence of irregularities in the mucosa surface properties (γ).

In this study, the application of heatmaps and SVM helped visualize gazing areas and reveal trends in diagnostic importance. However, it was evident that, with the increase in the number of subjects and coordinates of the gazing points, we face challenges in visualizing the decision boundary in SVM. Thus, further studies may need alternative strategies for capturing important areas inside the lesion.

This study presents new categories based on the nongenetic classification that extend beyond malignant tumors to other oral mucosal diseases. Notably, each category is characterized by a distinctive site that contributes to a correct diagnosis. Moreover, the trend toward worse prognosis in lesions with certain gross features aligns with previously reported cases [26,27,28,29,30].

The study findings have significant implications for future research and clinical practice. They provide a new perspective on the examination of oral mucosal diseases and the potential for early detection of malignancies [31,32]. Furthermore, the findings could be used as an educational tool, aiding in the training of clinicians and enhancing diagnosis. It will also improve the accuracy of photographic diagnosis of oral mucosal diseases when providing online medical care, enabling appropriate risk assessment.

This study has some limitations. Selection bias may have been introduced through the criteria for intraoral photographs and because most subjects were from a single institution, Osaka University Dental Hospital. Future research should gather a more diverse dataset, considering different lesion sizes, sites, degrees of diagnostic difficulty, and multiple institutions. Furthermore, we recommend exploring ways to conduct statistical analyses that account for these varying factors.

Extending our research to include normal and similar mucosal photographs could provide a more comprehensive understanding of the examination processes in regular dental checkups and potentially reduce overlooked diseases.

This study makes a significant contribution to the medical field by providing new insights into the use of gaze data for diagnosing oral mucosal diseases. However, more research is needed to validate our findings, improve the study limitations, and further explore the potential uses of gaze data in the future.

## 5. Conclusions

The utilization of visualization tools like heatmaps and SVM has significantly advanced our understanding of clinicians’ gaze patterns when assessing intraoral photographs. These tools have not only delineated the differences in examination strategies between skilled oral surgeons and general dentists but have also unraveled key insights into their diagnostic processes.

Moreover, this study paves the way for future research in gaze detection technology. By further exploring the intricate dynamics of gaze patterns in various medical specializations, we can unlock a deeper understanding of diagnostic reasoning and technique. This, in turn, could revolutionize training methods for medical professionals and refine diagnostic tools, contributing significantly to the field of medical diagnostics.

## Figures and Tables

**Table 1:** Demographic and professional characteristics of the study participants categorized as “Experience” and “Area of Expertise” (n = 78).

		Male	Female	Total
Experience	(i) Resident dentist	11	5	16
	(ii) 2–7 years	26	16	42
	(iii) 8–15 years	7	4	11
	(iv) ≥16 years	5	4	9
Area of expertise	(i) Initial training group	11	5	16
	(ii) Young oral surgery group (<7 years of clinical experience)	18	8	26
	(iii) Skilled oral surgery group (minimum 8 years of clinical experience)	8	5	13
	(iv) General dentist group (nonoral surgery specialty)	12	11	23

## Data Availability

The datasets generated and/or analyzed in this study have not been published due to ethical concerns regarding patient’s confidentiality but are available from the corresponding authors upon reasonable request.
